# Mangrove rare actinobacteria: taxonomy, natural compound, and discovery of bioactivity

**DOI:** 10.3389/fmicb.2015.00856

**Published:** 2015-08-20

**Authors:** Adzzie-Shazleen Azman, Iekhsan Othman, Saraswati S. Velu, Kok-Gan Chan, Learn-Han Lee

**Affiliations:** ^1^Jeffrey Cheah School of Medicine and Health Sciences, Monash University Sunway CampusSelangor, Malaysia; ^2^Division of Genetics and Molecular Biology, Faculty of Science, Institute of Biological Sciences, University of MalayaKuala Lumpur, Malaysia

**Keywords:** mangrove, rare actinobacteria, natural compounds, bioactivity, drug discovery

## Abstract

*Actinobacteria* are one of the most important and efficient groups of natural metabolite producers. The genus *Streptomyces* have been recognized as prolific producers of useful natural compounds as they produced more than half of the naturally-occurring antibiotics isolated to-date and continue as the primary source of new bioactive compounds. Lately, *Streptomyces* groups isolated from different environments produced the same types of compound, possibly due to frequent genetic exchanges between species. As a result, there is a dramatic increase in demand to look for new compounds which have pharmacological properties from another group of *Actinobacteria*, known as rare actinobacteria; which is isolated from special environments such as mangrove. Recently, mangrove ecosystem is becoming a hot spot for studies of bioactivities and the discovery of natural products. Many novel compounds discovered from the novel rare actinobacteria have been proven as potential new drugs in medical and pharmaceutical industries such as antibiotics, antimicrobials, antibacterials, anticancer, and antifungals. This review article highlights the latest studies on the discovery of natural compounds from the novel mangrove rare actinobacteria and provides insight on the impact of these findings.

## Introduction

Market demand for new drugs is extremely urgent and extensive due to common ailments and the fast spread of diseases, the development of new diseases with unknown causes, and the spread of antibiotic-resistant pathogens (Jiang et al., 2008). With the increasing advancement in science and technology, it is predicted that there would be a greater demand for new bioactive compounds synthesized by *Actinobacteria* from various sources including soil and marine.

Mangrove is a unique woody plant community of intertidal coasts in tropical and subtropical coastal regions. The mangrove forests are among the world's most productive ecosystems which improves coastal water, produces commercial forest products, supports coastal fisheries, and protects coastlines. Due to its properties of high salinity, strong winds, extreme tides, high temperature, anaerobic soils, and muddiness; little is known about the bacterial community living in the mangrove especially of *Actinobacteria* with the potential to produce bioactive metabolites.

*Actinobacteria* or *Actinomycetes* are Gram positive bacteria with high guanine (G) and cytosine (C) rations in their DNA (Goodfellow and Williams, 1983). According to Das et al. (2008), the name “*Actinomycetes*” was derived from two Greek words, “atkis” which means *ray*, and “mykes” which means *fungus;* and it has features of both bacteria and fungi. In the strict taxonomic sense, *Actinobacteria* are grouped with bacteria under the class “Schizomycetes” but limited to the order of Actinomycetales (Sivakumar et al., 2005; Gayathri and Muralikrishnan, 2013). *Actinobacteria* can be divided into two groups namely, *Streptomyces* and non-*streptomyces* or also known as rare actinobacteria.

Many studies have found that bioactive compounds of actinobacteria possess a wide range of biological activities. In the late Twentieth Century, some natural products produced by *Actinobacteria* had been used extensively in clinical settings as antibacterials, antifungals, anticancer, antitumor, and antiparasitics (Butler, 2004). It is important to discover new natural metabolites to handle the problem of antibiotic-resistant pathogens which are no longer susceptible to the antibiotics available. However, the recent search for the novel compounds always lead to the rediscovery of known compounds from the same species (Koehn and Carter, 2005).

Currently, the discoveries of new natural metabolites are focusing on non-*Streptomyces* or rare actinobacteria. Rare actinobacteria are always referred to as strains that are difficult to isolate and might correspond to the unmatched source of new natural metabolites (Baltz, 2006). The bioactivity studies of natural metabolites from the mangrove rare actinobacteria has become popular. Compounds discovered from the mangrove rare actinobacteria are uniquely structured and lead directly to the development of novel antibiotics that are effective against antibiotic-resistant pathogens (Lam, 2006).

## Mangrove

Mangrove swamps occupy about 181,000 km^2^ (Jusoff, 2013) or cover approximately 75% of the world's tropical and subtropical coastlines (Yang et al., 2013). Mangroves are mostly tropical trees that grow between the high spring tide mark in stable shores, and near mean sea levels (Holguin et al., 2001). According to Tomlinson (1986), a mangrove requires five criteria to be considered a “true or strict mangrove” namely, (1) complete fidelity to the mangrove environment; (2) plays a major role in the structure of the community and has the ability to form pure stands; (3) morphological specialization for adaptation to the habitat; (4) physiological specialization for adaptation to their habitat; and (5) taxonomic isolation from terrestrial relatives. Mangroves are thus a unique environment with highly productive ecosystems (global litterfall of 100 Tg C year^−1^) (Jennerjahn and Ittekkot, 2002). The important physical features of the mangrove that aid their survival in the boundary zone between ocean and land are adaptations for mechanical fixation in loose soil, specialized dispersal mechanisms, breathing roots and air exchange devices, and specialized mechanisms for dealing with excess salt concentration (Spalding et al., 1997).

Microbes constitute the largest pool of metabolic pathways on earth with potential biotechnological and environmental implications. In 1988, Alongi reported that in tropical mangroves, 91% of the total microbial biomass is bacteria and fungi, another 7% is algae, and 2% is protozoa (Alongi, 1988). The microbial diversity of mangrove ecosystems provides information on their ecological role and unique biotechnological potential in the field of agriculture, industry, medicine, and pharmaceuticals (Lageiro et al., 2007). The bacterial communities in mangrove environments were firstly determined by Yan et al. (2006) and Liang et al. (2007). Both studies used molecular cloning and sequencing approaches to understand the diversity of prokaryotes in these environments (Andreote, 2012).

The mangrove ecosystem is a largely unexplored source of *Actinobacteria* with the potential to produce active secondary metabolites (Hong et al., 2009). Although several studies on bacterial productivity and activity within the mangrove ecosystems were conducted, little is known about their genetic and metabolic diversity.

## Metagenomics of mangrove microbial communities: pyrosequencing and fosmid library

Molecular phylogenetic studies have shown that only a small number (~1%) of bacterial diversity present in environmental samples, are readily cultivable using conventional culture-based techniques (Yan et al., 2013). It is evident that more than 88% of isolates belong to four phyla known as *Proteobacteria, Firmicutes, Actinobacteria*, and *Bacteroidetes* (Nikolaki and Tsiamis, 2013). The uncultivated bacteria may serve as a prolific source of new bioactive compounds as they may possess a great diversity of enzymes to be easily adapted to different environmental conditions. Unlike other strategies used for novel enzyme and microorganism identification, the metagenomic method has clear advantages as an alternative to culture dependent methods. Metagenomics is a culture-independent genomic analysis of bacteria diversity contained in a sample, reducing difficulties related to conditions for bacteria cultivation (Handelsman, 2004). Metagenomics uses different methods ranging from the generation of short sequence reads by direct use of high-throughput sequencing technologies, to the construction and sequencing of large-insert DNA libraries (Reigstad et al., 2011).

Next generation sequencing (NGS) has significantly increased sequencing output via the use of extremely parallel sequencing (Staley et al., 2013). The advancement of the next-generation DNA sequencing technologies such as high-throughput pyrosequencing and Illumina platform, increases scientific interests in understanding the microbial diversity in different environments. The study of bacterial communities from soils (Jones et al., 2009), marine water (Brown et al., 2009), wastewater (Sanapareddy et al., 2009) and human microbiomes (NIH HMP Working Group et al., 2009) have been successfully conducted using the NGS approach. High-throughput pyrosequencing is a tool for the analysis of environmental microbiome, and reveal the taxonomic diversity of a specific environment at high resolution (Zhu et al., 2011). This method enables rapid estimation of bacterial diversity and furthermore, provides information about environmental interference. The NGS approach enables the discovery of novel enzymes and molecules with potential application in the biotechnology industry (Duan and Feng, 2010). The NGS technology is known to be costly for the assessment of huge numbers of environmental samples with high sequencing depths (Zhou et al., 2011). However, the advancement in NGS technology has enabled Illumina to dominates the market with 60% of the second generation sequencer installation that successfully reducing the cost with the used of reversible terminator-based sequencing by the synthesis chemistry, small, and less expensive, Illumina MiSeq platform (Niedringhaus et al., 2011).

The application of NGS allows the study of microbial diversity without culturing the bacteria hence, avoiding the loss of unculturable bacteria on laboratory media such as fastidious bacteria. The latest metagenome study on characterization of mangrove soil collected from Rantau Abang, Malaysia via NGS was done by Chan and Ismail (2015). In this study, they showed that the *Proteobacteria* (43.72%) was the dominant phyla followed by *Acidobacteria* (17.68%), *Firmicutes* (13.45%), *Actinobacteria* (4.55%), *Nitrospirae* (4.22%), *Planctomycetes* (3.06%), *Chloroflexi* (2.88%), *Verrucomicrobia* (2.69%), *Spirochaetes* (1.70%), *Chlamydiae* (1.32%), and *Bacteroidetes* (1.31%). However, the unclassified bacteria found in this study were clustered as Caldithrix, Haloplasmatales, and phototrophic bacteria. A study on the profile of bacterial community in different depths of soil in Sundarbans mangrove, India was done by Basak et al. (2015). The taxanomic analysis of 2746 species showed that they belong to 33 different phyla such as *Proteobacteria, Firmicutes, Chloroflexi, Bacteroidetes, Acidobacteria, Nitrospirae*, and *Actinobacteria*. The description of mangrove microbiology has been made on the basis of pyrosequencing, based on DNA extracted directly from the sediment of four distinct mangrove areas along the coast of the Sao Paulo State in Brazil (Andreote et al., 2012). The major bacterial phyla identified from this study was *Proteobacteria* (47.1—56.3%) followed by *Firmicutes* (10.5—13.8%), *Actinobacteria* (5.4—12.2%), *Bacteroidetes* (3.8—11.8%), and *Chloroflexi* (1.3—5.4%); and some minor phyla such as *Planctomytes, Cyanobacteria, Acidobacteria*, and Archaea. Furthermore, Andreote et al. (2012) compared the mangrove metagenomes with other related studies, which indicated that the phylum *Actinobacteria* was mostly found in estuarine sediments. The metagenomic data from Thompson et al. (2013) showed similar predominant bacterial phyla in two different mangrove areas in Brazil, namely Rio de Janeiro and Bahia; were *Proteobacteria* (57.8% and 44.6%), *Firmicutes* (11% and 12.3%), and *Actinobacteria* (8.4% and 7.5%). Furthermore, the *Actinobacteria, Fibrobacteres/Acidobacteria*, and *Firmicutes* group were predominantly represented in the Bahia mangrove area. Gomes et al. (2010) performed an educated measurement of rhizosphere microbial population in comparison to bulk sediments samples. The major bacterial order discovered were *Burkholderiales, Caulobacterales*, and *Rhizobials* while the major bacterial phyla discovered were *Acidobacteria, Actinobacteria*, and *Verrucomicrobia*. The formation of metagenomic libraries using fosmid library become a method of choice for exploring environmental microbial communities that are difficult to culture and maintain.

## Actinobacteria

Under the domain Bacteria, the *Actinobacteria* is one of the most distributed phyla among the 30 major lineage including 5 subclasses (www.bacterio.net). According to Zhi et al. (2009), under the class *Actinobacteria*, there are five orders known as *Acidimicrobiales, Rubrobacterales, Coriobacterales, Bifidobacteriales*, and *Actinomycetales*. However, the recent list showed that there are five new orders under *Actinobacteria* known as *Nitriliruptorales* (Sorokin et al., 2009), *Solirubrobacterales* (Reddy and Garcia-Pichel, 2009), *Thermoleophilales* (Reddy and Garcia-Pichel, 2009), *Euzebyales* (Kurahashi et al., 2010), and *Gaiellales* (Albuquerque et al., 2011), making a total of ten orders (www.bacterio.net). In nature, *Actinobacteria* are also known as saprophyte soil inhabitants (Naik et al., 2013), and one of the dominant colonizers in soils (McCarthy and Williams, 1992; Heuer et al., 1997; Lee et al., 2012). *Actinobacteria* are Gram positive bacteria with a high G+C content in their DNA, ranging from 51% in some corynebacteria, to more than 70% in *Streptomyces* and *Frankia* except for *Tropheryma whipplei;* an obligate pathogen which contains less than 50% G+C (Marco et al., 2007).

*Actinobacteria* have a variety of morphologies, from rod-coccoid (e.g., *Arthrobacter* spp.) or coccoid (e.g., *Micrococcus* spp.), to permanent or highly differentiated branch mycelium (e.g., *Streptomyces* spp.), or fragmenting hyphal forms (e.g., *Nocardia* spp.) (Atlas, 1997). They also show various physiological and metabolic properties, such as the production of extracellular enzymes and a variety of secondary metabolites such as potent antimicrobial agents (Lee et al., 2014a; Olano et al., 2014). The member of genus *Streptomyces* species displayed rare development characteristics with the formation of aerial mycelium with spore (Lee et al., 2014b; Ser et al., 2015), whereas certain mycobacterium exhibited a persistent non-replicating state. In common laboratory media, *Actinobacteria* tends to grow slowly as branching filaments and have the ability to produce motile spores (Nanjwade et al., 2010). *Actinobacteria* can be divided into two main groups; the *Streptomyces*, representing the dominant species in the group; and the rare actinobacteria.

## Rare actinobacteria

In their natural habitat, *Streptomyces* are the dominant species of *Actinobacteria*. Rare actinobacteria are relatively difficult to isolate, culture, and maintain; due to the difficulties in maintaining and mimicking their natural environment. Rare actinobacteria have been considered as *Actinobacteria* with lower isolation rates compared to *Streptomyces* strains using conventional isolation methods, due to the requirement of using appropriate isolation procedures and to apply different selection conditions (Khanna et al., 2011). Until September 2010, there are approximately 220 genera of rare actinobacteria reported (Tiwari and Gupta, 2013). More evidences have shown that increasing numbers of rare actinobacteria have been discovered from different sources, thus indicating that rare actinobacteria are widely distributed in the biospheres. Even though soils and water are their major habitat, they are also isolated from different environments such as the deep ocean, desert, mangroves, plants, caves, volcanic rocks, and stones (Groth et al., 1999). In natural ecosystems, many chemical, biological, and physical elements; affect their diversity as the environmental factors such as pH, humus content, soils type, temperature, and salinity affect their distribution (Hayakawa, 2008).

The isolation of rare actinobacteria could definitely increase the chances of discovering potentially novel bioactive compounds, therefore it is vital to be equipped with better knowledge of their diversity and distribution in the environment; in order to facilitate the isolation of these strains using an efficient approach (Hong et al., 2009; Tiwari and Gupta, 2012; Lee et al., 2014a). According to Jose and Jebakumar (2013), rare actinobacteria are distributed among 24 genera: *Actinomadura, Actinoplanes, Amycolatopsis, Actinokineospora, Acrocarpospora, Actinosynnema, Catenuloplanes, Cryptosporangium, Dactylosporangium, Kibdelosporangium, Kineosporia, Kutzneria, Microbiospora, Microtetraspora, Nocardia, Nonomuraea, Planomonospora, Planobispora, Pseudonocardia, Saccharomonospora, Saccharopolyspora, Saccharothrix, Streptosporangium*, and *Spirilliplanes*.

### Rare actinobacteria of mangrove origin

The mangrove ecosystem is among the world's most productive environment, and it is a huge unexplored source of *Actinobacteria* with a high potential to produce active secondary metabolites (Hong et al., 2009). Indeed relatively little is known about the rare actinobacteria living in the mangrove ecosystem. Recently the mangrove ecosystem has become popular for novel strains and novel bioactive compound discovery.

A few reports from different geographical locations globally have described the diversity and isolation of novel rare actinobacteria in different mangrove habitats. In 2008, Eccleston et al. reported the occurrence of *Microsmonospora* from the Sunshine Coast in Australia (Eccleston et al., 2008). Xie et al. (2006) and Huang et al. (2008) reported the isolation of a rifamycin-producing *Micromonospora* from mangrove in South China Sea. Different genera such as *Brevibacterium, Dermabacter, Kytococcus, Microbacterium, Nesterenkonia*, and *Rothia* were isolated from mangrove sediments in Brazil (Dias et al., 2009b). In China, a number of rare actinobacteria including *Actinomadura, Isoptericola, Microbispora, Nocardia, Nonomuraea*, and *Rhodococcus;* were isolated from mangrove soils and plants (Hong et al., 2009). Results from Ara et al. (2013) showed that 17 different genera of rare actinobacteria were identified from a total of 241 isolates. It is reported that the predominant genus detected was *Micromonospora* in both mangrove and medicinal plant rhizosphere soil samples in Dhaka, Bangladesh. This result was in line with other studies (Cross, 1981; Jiang and Xu, 1996; Hatano, 1997; Ara et al., 2002) which exhibited *Micromonospora* was the predominant genus in isolates from wet soil. A study done by Lee et al. (2014a) on the diversity of actinobacteria from a Malaysian mangrove forest showed that 40.2% of isolates were rare actinobacteria and the predominant genus was *Mycobacterium*. The study also discovered other genera of rare actinobacteria, such as *Leifsonia, Streptacidiphilus, Sinomonas*, and *Terrabacter*, which were not commonly found in mangrove environment. In this study, the understanding of rare actinobacteria diversity led to the discovery of novel genus and species strains, such as “*Microbacterium mangrovi* sp. nov.” (Lee et al., 2014c), “*Mumia flava* gen. nov., sp. nov.” (Lee et al., 2014d) and *Sinomonas humi* sp. nov.” (Lee et al., 2015) isolated from a mangrove forest in Tanjung Lumpur, Malaysia. The discoveries of novel rare actinobacteria from mangrove environments, from 2001 until 2015 are listed in Table 1.

**Table 1:** **Novel mangrove rare actinobacteria discovered between the years 2001 and 2015**.

**Genus**	**Strain name and designation**	**Sources**	**References**
*Agromyces*	*Agromyces luteolus*, 8^T^, *Agromyces rhizospherae*, 14^T^, *Agromyces brachium*, 65^T^	Rhizosphere of mangroves in the estuary of the Shiira River, Iriomote Island, Japan	Takeuchi and Hatano, 2001
*Asanoa*	*Asanoa iriomotensis*, TT 97-02^T^	Soil at roots of the mangrove *Bruguiera gymnorrhiza*	Tamura and Sakane, 2005
*Polymorphospora*	*Polymorphospora rubra*, TT 97-42^T^	Soil near the roots of *Bruguiera gymnorrhiza* and *Sonneratia alba* at the River Shiira, Iriomote Island, Okinawa, Japan	Tamura et al., 2006
*Nonomuraea*	*Nonomuraea Maheshkhaliensis*, 16-5-14^T^	Mangrove forest in Maheshkhali, Cox's Bazar, Bangladesh	Ara et al., 2007
*Micromospora*	*Micromonospora pattaloongensis*, TJ2-2^T^	Mangrove forest in Pattaloong Province, Thailand	Thawai et al., 2008
*Micromonospora*	*Micromonospora rifamycinica*, AM105^T^	Mangrove sediment collected from the South China Sea	Huang et al., 2008
*Actinomadura*	*Actinomadura maheshkhaliensis*, 13-12(50)^T^	Mangrove rizhosphere soils of Maheshkhali, Bangladesh	Ara et al., 2008
*Verrucosispora*	*Verrucosispora lutea*, YIM 013^T^	Shenzhen Futian Mangrove	Liao et al., 2009
*Demequina*	*Demequina salsinemoris*, KV-810^T^	Mangrove soil from a southern island in Japan	Matsumoto et al., 2009
*Sphaerisporangium*	*Sphaerisporangium krabiense*, A-T 0308^T^	Tropical mangrove forest soil from Thailand	Suriyachadkun et al., 2011
*Isoptericola*	*Isoptericola chiayiensis*, 06182M-1^T^	Mangrove soil from Chiayi Country in Taiwan	Tseng et al., 2011
*Micromonospora*	*Micromonospora rhizosphaerae*, 211018^T^	Mangrove *Excocaria agallocha* rhizosphere soil	Wang et al., 2011a
*Nonomuraea*	*Nonomuraea wenchangensis*, 210417^T^	Mangrove rhizosphere soil	Wang et al., 2011b
*Jishengella*	*Jishengella endophytica*, 202201^T^	Acanthus illicifolius root from the mangrove reserve zone in Hainan, China	Xie et al., 2011
*Asanoa*	*Asanoa hainanensis*, 210121^T^	Rhizosphere soil of the mangrove fern *Acrostichum speciosum*	Xu et al., 2011
*Microbispora*	*Microbispora hainanensis*, 211020^T^	Rhizosphere mangrove soil of *Exoecaria agallocha*, Hainan, China	Xu et al., 2012
*Lysinimicrobium*	*Lysinimicrobium mangrovi*, HI08-69^T^	Rhizosphere mangrove soils from Iriomote Island, Japan	Hamada et al., 2012
*Verrucosispora*	*Verrucosispora qiuiae*, RtIII47^T^	Mangrove swamp in Sanya, Hainan Province, China	Xi et al., 2012
*Actinomadura*	*Actinomadura sediminis*, YIM M 10931 ^T^	Mangrove sediments from Dugong Creel, Little Andaman, India	He et al., 2012
*Agromyces*	*Agromyces indicus*, NIO-1018^T^	Mangrove sediment of the Chorao Island, Goa, India	Dastager et al., 2012
*Micromonospora*	*Micromonospora haikouensis*, 232617^T^	Composite mangrove sediment from Haikou, China	Xie et al., 2012
*Micromonospora*	*Micromonospora maritime*, D10-9-5^T^	Mangrove soil in Samut Sakhon province, Thailand	Songsumanus et al., 2013
*Micromonospora*	*Micromonospora avicenniae*, 268506^T^	Root of *Avicennia marina* collected at mangrove forest in Wengchang, Hainan province, China	Li et al., 2013a
*Actinoallomurus*	*Actinoallomurus acanthiterrae*, 2614A723^T^	Rhizosphere soil of mangrove plant *Acanthus ilicifolius* from Touyuan, Wenchang, Hainan province, China	Tang et al., 2013
*Micromonospora*	*Micromonospora sonneratiae*, 274745^T^	Root of *Sonneratia apetala* from mangrove forest in Sanya, Hainan province, China	Li et al., 2013b
*Micromonospora*	*Micromonospora wenchangensis*, 2602GPT1-05^T^	Composite mangrove soil from Wenchang, Hainan province, China	Ren et al., 2013
*Microbacterium*	*Microbacterium mangrovi*, MUSC 115^T^	Mangrove soil of Tanjung Lumpur river, State of Pahang, Malaysia	Lee et al., 2014c
*Mumia*	*Mumia flava*, MUSC 201^T^	Mangrove soil of Tanjung Lumpur river, State of Pahang, Malaysia	Lee et al., 2014d
*Sinomonas*	*Sinomonas humi*, MUSC 117^T^	Mangrove soil of Tanjung Lumpur river, State of Pahang, Malaysia	Lee et al., 2015

## Natural compounds from rare actinobacteria

The discovery of the first antibiotic, penicillin, in 1929 heralded the era of antibiotics. Streptomycin was later isolated from *Streptomyces griseus* by Waksman in 1943, while vancomycin was discovered in 1953 as a metabolite of the rare actinobacteria strain, *Amycolatopsis orientalis* (Mahajan and Balachandran, 2012). The discovery of various useful antibiotics from rare actinobacteria contributes as an important part in the discovery of novel natural products (Ara et al., 2013). Researchers understand the role of microorganisms to provide rich sources of useful natural products for clinical purposes (Subramani and Aalbersberg, 2012). The demand for new drugs in the world is extremely urgent and extensive due to the fast spread of diseases, common ailments, and the fast spread of antibiotic-resistant pathogens (Jiang et al., 2008). The continuous advancement in science and technology would enable the accelerated discovery of useful bioactive compounds from prolific producers such as the *Actinobacteria*.

As of the year 2002, over 10,000 bioactive compounds (45% of all microbial metabolites) were produced by filamentous *Actinobacteria*, out of which 7600 (75%) were derived from *Streptomyces*, and 2500 (25%) from rare actinobacteria such as *Micromonospora, Actinomadura*, and *Streptoverticillium* (Bérdy, 2005). *Actinobacteria* are able to produce different types of secondary metabolites due to the function of various genes such as the non-ribosomal polyketides synthase (NRPS) and polyketide synthase (PKS) (Salomon et al., 2004; Lee et al., 2014a). In spite of the tremendous success of the past in screening for useful secondary metabolites, the chance of finding new biologically active molecules from *Streptomyces* has decreased (Fenical et al., 1999). As some *Streptomyces* strains from different environments produced the same types of compounds, possibly due to frequent genetic exchange between species (Bredholt et al., 2008; Freel et al., 2011). As a result, there is a dramatic increase in demand to look for new pharmacological important compounds.

The advancement of new selective techniques enables the isolation and screening of rare actinobacteria, which could lead to the discovery of new and useful bioactive compounds. The discovery of rare actinobacteria has enlarged the range and diversity of genetic resources available for biotechnological exploitation. It is apparent that the discovery of novel rare actinobacteria can be expected to provide new bioactive compounds (Lazzarini et al., 2000; Donadio et al., 2002; Takahashi and Ômura, 2003; Bérdy, 2005; Hong et al., 2009; Lee et al., 2014a).

The review mainly focuses on five different genera with the species found from the mangrove environment namely, *Micromonospora, Jishengella, Salinispora, Saccharopolyspora*, and *Nocardiopsis*. They are all proven to have valuable sources of potential metabolites and besides *Streptomyces*; they have proven to be valuable sources of potentially useful bioactive metabolites.

### Micromonospora

*Micromonospora* is a genus of *Actinobacteria* from the *Micromonosporaceae* family. This genus was first isolated by Ørskov in 1923, is Gram positive, aerobic, and chemo-organotrophic (Ann and Maria, 2009). Currently, this genus contains 59 species and 7 subspecies (http://www.bacterio.net/). Redenbach et al. (2000) revealed that *Micromonospora chalcea* DSM 43026 contained a linear chromosome with a size of 7.7 Mb. Hirsch et al. (2013) reported the complete genome sequence of *Micromonospora* strain L5 isolated from nodules of *Casuarina equisetifolia* trees in Mexico. The strain *Micromonospora* strain L5 has a genome size of 6,907,073 bp, with 6332 predicted open reading frames (ORFs) and the GC content of 72.86%. The genome was sequenced using a combination of Illumina and 454 technologies at the Joint Genome Institute (JGI). According to Hirsch and Valdés (2009), the genome size of *Micromonospora aurantica* is 6.97 Mb and contains 88.64% of GC. However, both genomes of *Micromonospora aurantianca* ATCC 37029 and *Micromonospora* strain L5 have not been annotated as yet.

**Figure 1:**
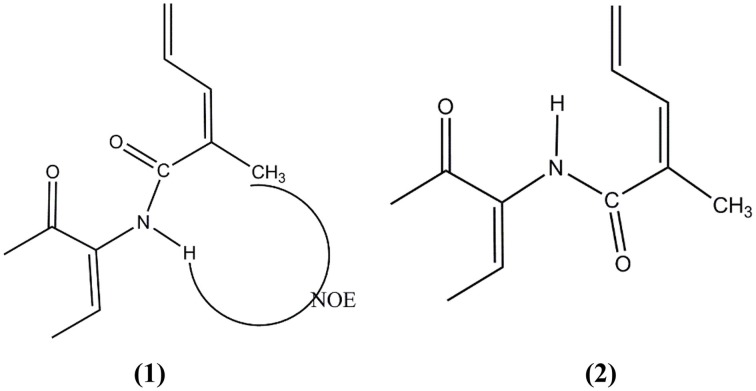
**The structure of rifamycin S (1) and the geometric isomer of rifamycin S (2)**.

Currently, some *Micromonospora* from the mangrove environment has produced useful bioactive compounds for studies on drug discovery. *Micromonospora rifamycinica* AM105 was isolated from mangrove sediments from Hainan Island, South China Sea (Huang et al., 2009). The antimicrobial activity against methicillin-resistant *Staphylococcus aereus* (MRSA) using secondary metabolite extracts has led to the discovery of two antibiotic compounds (Figure 1), namely rifamycin S (**1**) and the geometric isomer of rifamycin S (**2**). There are different types of rifamycin, depending on its chemical structure such as rifamycin B, O, S, and SV. Rifamycin S has higher activity once it reduces to form rifamycin SV in aqueous solutions (Zhang, 2003). Also the antibacterial activity from a combination of rifamycin S and the geometric isomer of rifamycin S from *Micromonospora rifamycinica* AM105 was higher than rifamycin SV. This finding suggests that the antibacterial activity of the geometric isomer of rifamycin S was higher than rifamycin SV, and the antimicrobial activity of rifamycin SV was higher than rifamycin S.

**Figure 2:**
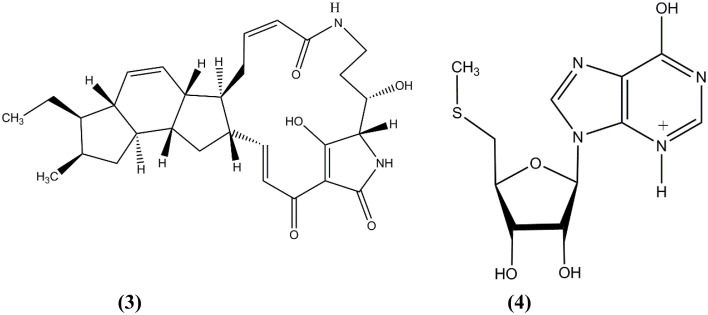
**The structure of butremycin (3) and protonated aromatic tautomer of 5′-methylthioinosine (MTI) (4)**.

Kyeremeh et al. (2014) discovered an actinobacterial strain, *Micromonospora* sp. K310, from Ghanaian mangrove river sediment. The spectroscopy study of this strain led to the discovery of two new compounds (Figure 2), butremycin (**3**) (the (3-hydroxyl) derivative of ikarugamycin) and protonated aromatic tautomer of 5′-methylthioinosine (MTI) (**4**). The antibacterial activity for butremycin showed it was not potent enough to kill pathogens such as *Staphylococcus aureus* ATCC 25923, *Escherichia coli* ATCC 25922, and clinical isolates of methicillin-resistant *Staphylococcus aureus* (MRSA) strains. The antimicrobial activity of the protonated aromatic tautomer of 5′-methylthioinosine showed no antibacterial activity against these pathogens.

**Figure 3:**
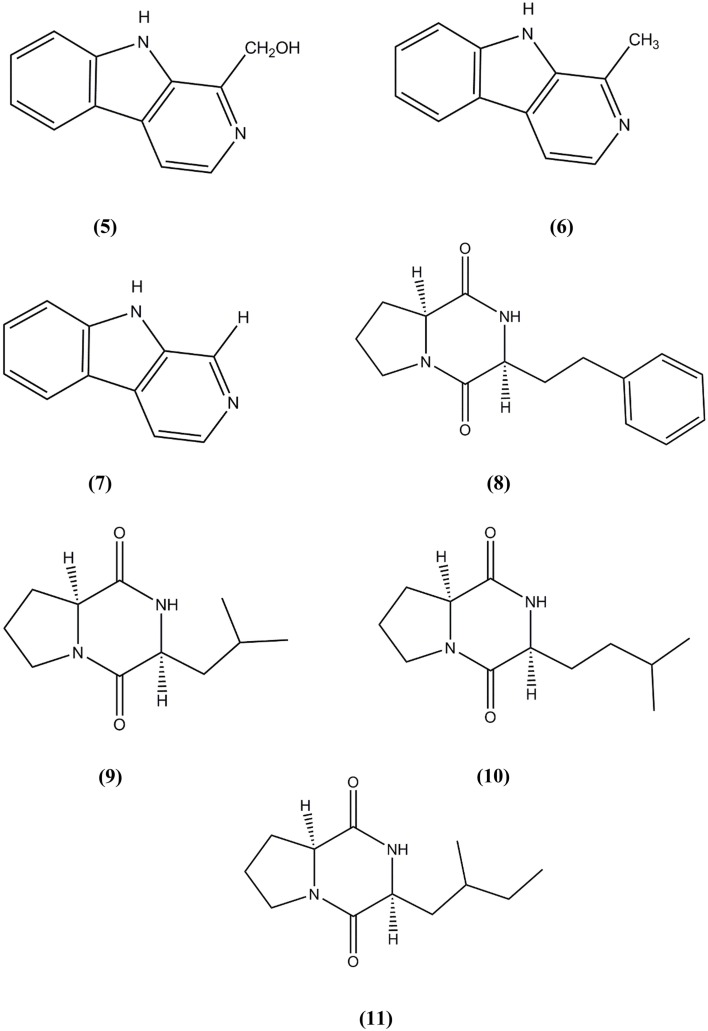
**The structure of 3-hydroxymethyl-β-carboline (5), 3-methyl-β-carboline (6), β-carboline (7), Cyclo-(L-Pro-L-Phe) (8), and Cyclo-(L-Pro-L-Leu) (9), Cyclo-(L-Pro-L-Ile) (10), and Cyclo-(L-Pro-L-Val) (11)**.

*Micromonospora* sp. M2DG17 was isolated from composite mangrove sediment in Haikou, China (Huang et al., 2011). The utilization of bioassay-guided fractionation enabled 5 compounds to be identified for the first time from the *Micromonospora* species along with two other compounds (Figure 3). The five compounds were identified as 3-hydroxymethyl-β-carboline (**5**), 3-methyl-β-carboline (**6**), β-carboline (**7**), Cyclo-(L-Pro-L-Phe) (**8**), and Cyclo-(L-Pro-L-Leu) (**9**) and the other two known compounds were Cyclo-(L-Pro-L-Ile) (**10**) and Cyclo-(L-Pro-L-Val) (**11**). An antitumor study using 3-methyl-β-carboline resulted in weak inhibitory on human colon carcinoma; HCT 116 cell lines (Huang et al., 2011).

### Jishengella

A novel genus, *Jishengella*, proposed by Xie et al. (2011); is one of the rare actinobacteria within the *Micromonosporaceae* family. *Jishengella* is the genus name given by a Chinese microbiologist, Jisheng Ruan. Currently, the genus consists of one species identified as *Jishengella endophytica* 202201^T^ (Xie et al., 2011).

Strain *Jishengella endophytica* 161111 has been able to produce a new alkaloid identified as 2-(furan-2-yl)-6-(2*S*,3*S*,4-trihydroxybutyl) pyrazine (**12**) (Wang et al., 2014). Extraction from the *Jishengella endophytica* 161111 fermentation broth resulted in the identification of three pyrazine derivatives (**12, 13, 14**), four pyrrololactones (**15, 16, 17, 18**), three β-carbolines (**19, 20, 21**), 1*H*-indole-3-carboxaldehyde (**22**), 2-hydroxy-1-(1*H*-indol-3-yl) ethanone (**23**), and 5-(methoxymethyl)-1*H*-pyrrole-2-carbaldehyde (**24**) (Wang et al., 2014). All compounds found in *Jishengella endophytica* 161111 are shown in Table 2.

**Table 2:** **Natural compounds discovered from *Jishengella endophytica* 161111**.

**Compounds**	**References**
**PYRAZINE DERIVATIVES**	
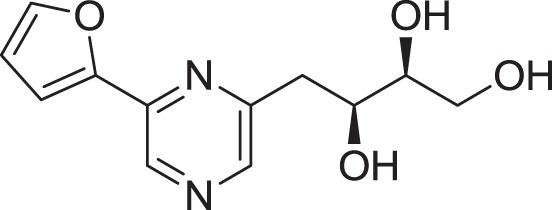	
2-(furan-2-yl)-6-(2*S*,3*S*,4-trihydroxybutyl) pyrazine **(12)**	Wu et al., 2007
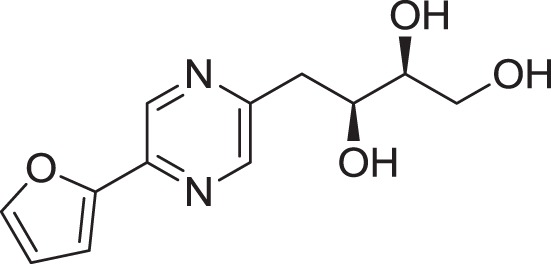	
2-(furan-2-yl)-5-(2*S*,3*S*,4-trihydroxybutyl) pyrazine **(13)**	Wu et al., 2007
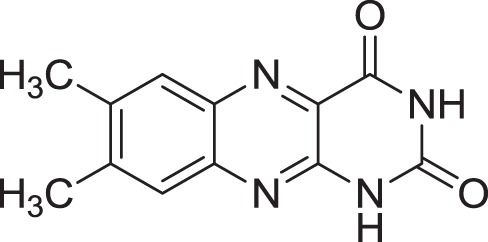	
Lumichrome **(14)**	Ding et al., 2009
**PYRROLOLACTONES**	
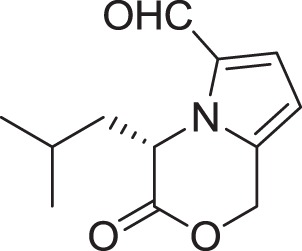	
(*S*)-4-isobutyl-3-oxo-3,4-dihydro-1*H*-pyrrolo[2,1-*c*][1,4]oxazine-6-carbaldehyde **(15)**	Sannai et al., 1982
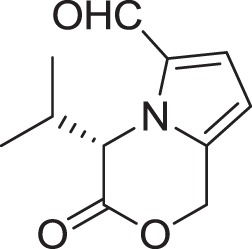	
(*S*)-4-isopropyl-3-oxo-3,4-dihydro-1*H*-pyrrolo [2,1-*c*][1,4]oxazine-6-carbaldehyde **(16)**	Sannai et al., 1982
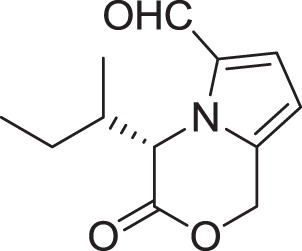	
(4*S*)-4-(2-methylbutyl)-3-oxo-3,4-dihydro-1*H*-pyrrolo [2,1-*c*][1,4]oxazine-6-carbaldehyde **(17)**	Sannai et al., 1982; Tressl et al., 1995
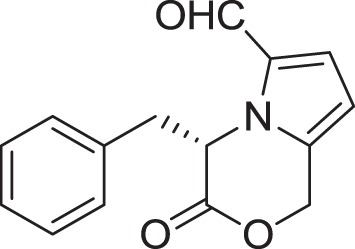	
(*S*)-4-benzyl-3-oxo-3,4-dihydro-1*H*-pyrrolo[2,1-*c*] [1,4]oxazine-6-carbaldehyde **(18)**	Jeric et al., 2000
**β-CARBOLIN**	
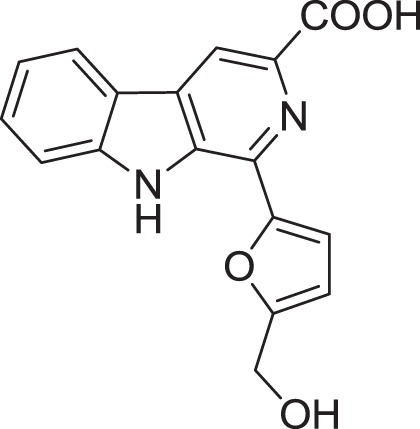	
Flazin **(19)**	Su et al., 2002
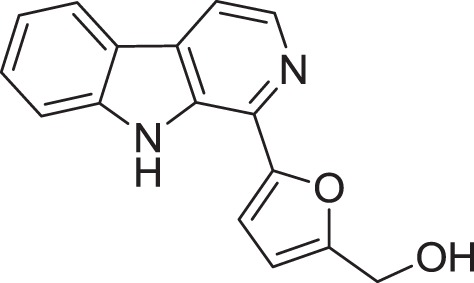	
Perlolyrine **(20)**	Dassonneville et al., 2011
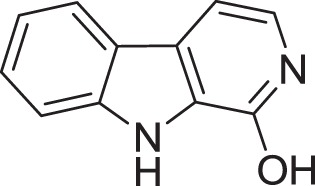	
1-hydroxy-β-carboline **(21)**	Jiao et al., 2010
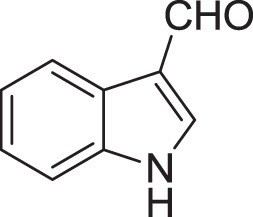	
1*H*-indole-3-carboxaldehyde **(22)**	Yang and Cordell, 1997; Ashour et al., 2007
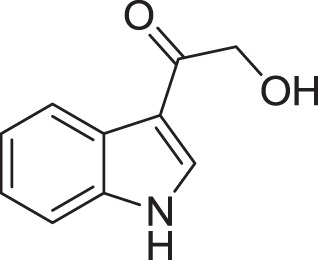	
2-hydroxy-1-(1*H*-indol-3-yl) ethanone **(23)**	Yang and Cordell, 1997; Ashour et al., 2007
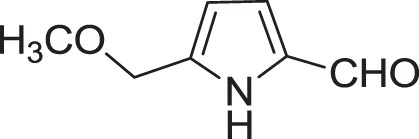	
5-(methoxymethyl)-1*H*-pyrrole-2-carbaldehyde **(24)**	Don et al., 2005

All compounds extracted from *Jishengella endophytica* 161111 were tested for antivirus effects on influenza A (H1N1) virus using the cytopathic effect (CPE) inhibition assay adapted from Grassauer et al. (2008) and Hung et al. (2009). Results showed that compounds **14, 20, 21**, and **22** displayed moderate anti-H1N1 activity (Wang et al., 2014). Furthermore, all compounds were tested for cytotoxic effects as described in Mosmann (1983). The study showed that only compounds **14, 20, 21**, and **22;** demonstrated weak cytotoxic activity against Madin-Dady canine kidney (MDCK) normal cells.

The β-carboline alkaloids are one of the largest groups of natural and synthetic indole alkaloids that have a common tricyclic pyrido [3,4-b] indole ring structure (Allen and Holmstedt, 1980). Originally, this compound was isolated from the plant, *Peganum harmala* (Zygophillaceae) located in the Middle East, India, and North Africa. It was used as an effective traditional herbal drug for its abortifacient effect, and emmanagogs (Moloudizargari et al., 2013). A study done by Wang et al. (2014) showed that the simple β-carboline isolated from *Jishengella endophytica* 161111 was able to kill the H1N1 virus. The unsubstitued H-3 is important in anti-H1N1 activity of this kind of compounds.

### Salinispora

*Salinispora* is a genus within the family *Micromonosporaceae* that was isolated from marine sediment in 2005 (Maldonado et al., 2005). *Salinispora* is an aerobic and Gram positive bacterium that formed extensive branched substrate hyphae with 0.25–0.5 μm in diameter. It is the first *Actinobacteria* that requires sea water to grow (Maldonado et al., 2005). Currently, the genus *Salinispora* consists of *three* species, namely *Salinispora arenicola, Salinispora tropica*, (Maldonado et al., 2005), and *Salinispora pacifica* (Jensen and Mafnas, 2006).

Udwary et al. (2007) reported the first complete genome of rare actinobacteria, *Salinispora tropica* CNB-440. This strain consists of 5,183,331 bp in size, which is approximately 3 Mbp smaller than the genome of *Streptomyces* (Bull and Stach, 2007). Analysis on the secondary natural product gene clusters showed that *Salinispora tropica* contains approximately 9.9% of the genomes dedicated to secondary metabolism, which is greater than previous *Streptomyces coelicolor* (≈8%) as well as other naturally-producing *Actinobacteria* (Udwary et al., 2007). The genome sequence of *Salinispora* revealed a great number of novel biosynthetic gene clusters, and based on the analysis; these findings confirms that this genus is one of the important sources of novel drug-like molecules. According to Bull and Stach (2007), 15 secondary metabolite gene clusters have been identified in *Salinispora tropica*.

**Figure 4:**
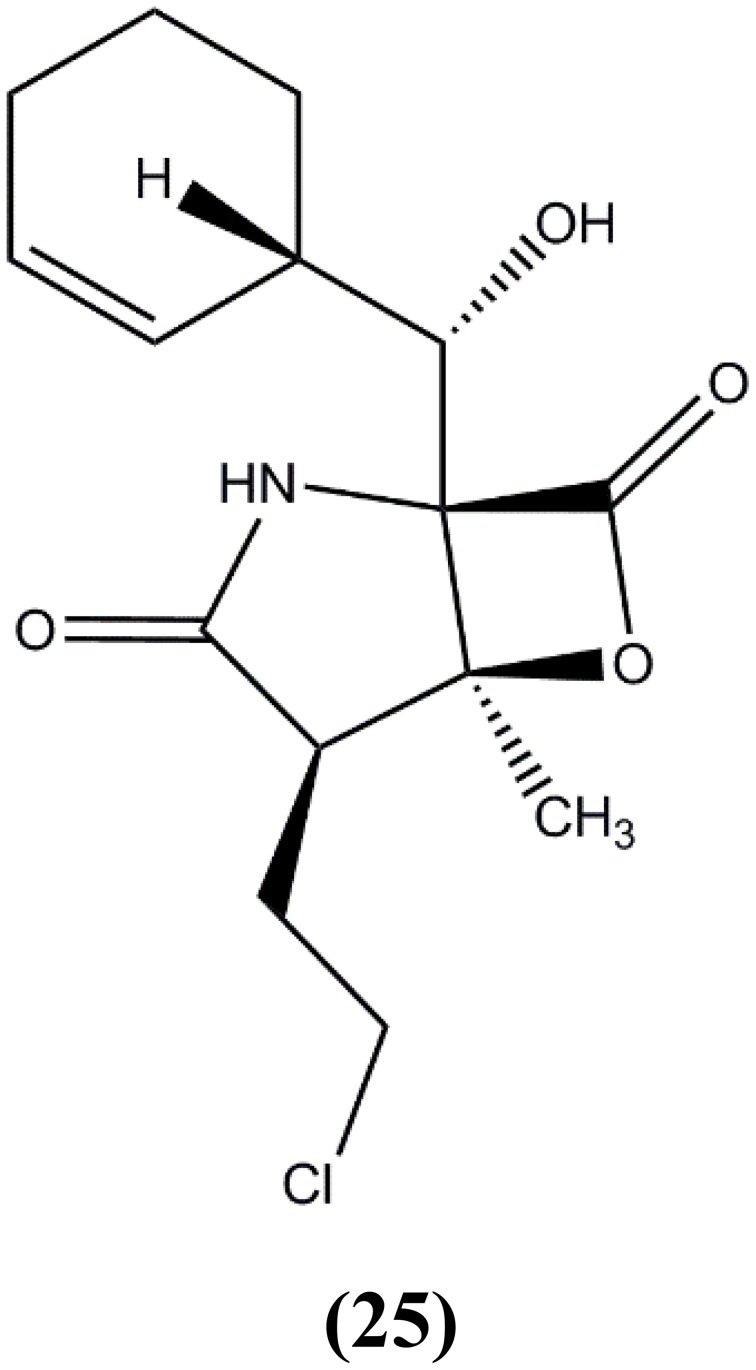
**The structure of Salinosporamide A (25)**.

A significant feature of *Salinispora tropica* is its ability to produce structurally unique secondary metabolites. Feling et al. (2003) reported that the *Salinispora tropica* CNB-392 isolated from a mangrove area in Chub Cay, Bahamas produced a unique metabolite named salinosporamide A (**25**) (Figure 4) attached with γ-lactam-β-lactone bicyclic core with *clasto*-lactacystin-β-lactone (also called as omuralide), a metabolite discovered from terrestrial *Streptomyces* sp. The study showed that this compound was able to kill different degrees of cancer cells and possessed strong inhibition of the 20S proteasome. Salinosporamide A was tested for the effects on proteasome function due to its structural relationship to omuralide. Results exhibited that Salinosporamide A was 35 times more potent compared to omuralide. Currently, the Salinosporamide A is in phase I human clinical trials as a potential anticancer drug (Fenical et al., 2009).

### Saccharopolyspora

The genus *Saccharopolyspora* was discovered in 1974 by Lacey and Goodfellow from heated sugar cane bagasse (Guan et al., 2011). At the time of writing, the genus *Saccharopolyspora* contained 26 species and 3 subspecies isolated from various sources (http://www.bacterio.net/). The first complete genome sequence of the *Saccharopolyspora* strain was reported by Oliynyk et al. (2007). The genome size of *Saccharopolyspora erythraea* is about 8.3 Mbp predicted to encode 7264 genes. *S. erythraea* genome contains at least 25 gene clusters for the production of known or predicted secondary metabolites. The draft genome of *Saccharopolyspora spinosa* NRRL 18395 was reported by Pan et al. (2011) which consists of approximately 8.6 Mbp with a GC content of 67.94%. The latest draft genome of *Saccharopolyspora rectivirgula* was reported by Pettersson et al. (2014). The draft genome of this strain consists of 182 contigs totaling 3,977,051 bp with a GC content of 68.9%.

**Figure 5:**
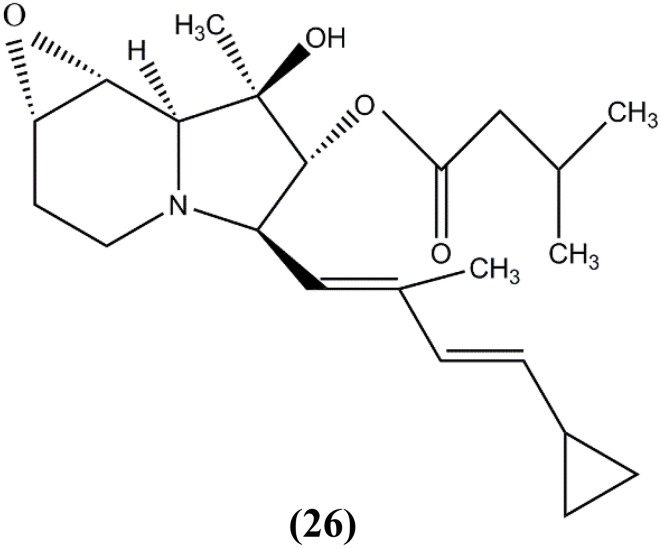
**The structure of JBIR-102 (26)**.

Izumikawa et al. (2012) isolated *Saccharopolyspora* sp. RL78 from mangrove soil in Nosoko, Ishigaki Island, Okinawa Prefecture, Japan. They discovered a new cyclizidine analog named JBIR-102 (**26**) (Figure 5), which exhibited cytotoxic activity against HeLa cells and human malignant pleural mesothelioma cell line, ACC-MESO-1.

### Nocardiopsis

*Nocardiopsis* is a genus of the family *Nocardiopsaceae* discovered in 1976 by Meyer. The bacterium contains fragmenting mycelium and a cell wall containing *meso*-diaminopimelic acid with no diagnostically important carbohydrates (Li et al., 2012). The genus *Nocardiopsis* contains 45 species and 5 subspecies with validly published names (http://www.bacterio.net/). The first report on genome sequence of genus *Nocardiopsis* was reported by Sun et al. (2010). Members of *Nocardiopsis dassonvillei* have been isolated from a variety of natural habitats such as soils and marine sediments, plant, animal material, and human patients. According to Sun et al. (2010), the genome size of *Nocardiopsis dassonvillei* is 6,543,312 bp with a 73% GC content, and 3.3% of gene cluster is for secondary metabolite biosynthesis, transport, and catabolism. The latest complete whole genome sequence of genus *Nocardiopsis* was announced by Qiao et al. (2012). *Nocardiopsis alba* strain isolated from honeybee guts contains 5,848,211 bp with a GC content of 69.65%. Based on the Antibiotics and Secondary Metabolites Analysis Shell (antiSMASH), 18 biosynthetic gene clusters were identified and at least 8 clusters were predicted for production of known antibiotics and secondary metabolites.

**Figure 6:**
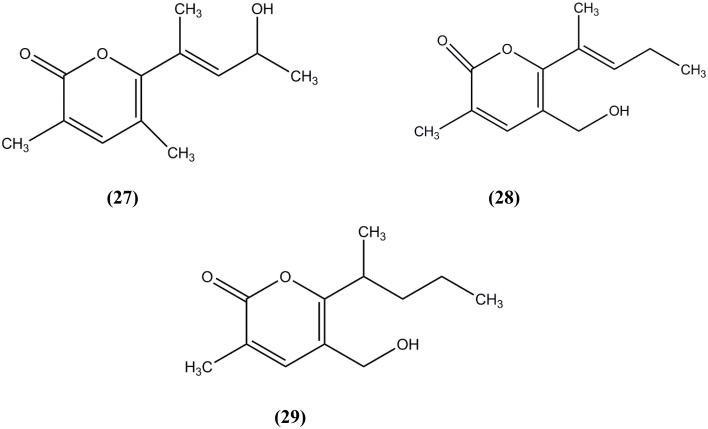
**The structure of Nocardiatones A (27), B (28), and C (29)**.

*Nocardiopsis* species were known to produce bioactive metabolites. Researchers discovered *Nocardiopsis* species were able to produce griseusin (Sun et al., 1991), apoptolidin (Kim et al., 1997), methylpendolmycin (Li et al., 2007a), thiopeptide (designated TP-1161) (Gandhimathi et al., 2009), lipopeptide biosurfactant (Ding et al., 2010), and napthhospironone A (Engelhardt et al., 2010). Three new 2-pyranone derivatives known as Nocardiatones A (**27**), B (**28**), and C (**29**) (Figure 6) were discovered from novel mangrove endophytic, *Nocadiopsis* sp. A00203 (Lin et al., 2010). This species was isolated from the leaves of *Aegiceras corniculatum* (Myrsinaceae). The structures of all compounds were examined using spectroscopic and mass-spectrometric analyses. The analysis of the anticancer activity of the compound was performed using HeLa cells and only Nocardiatones A showed weak cytotoxic activity against HeLa cells. No inhibition was observed, when these compounds were tested on *Escherichia coli, Bacillus subtilis, Staphylococcus aureus*, and yeasts.

## Conclusion

The discovery of novel species and novel natural compounds from the mangrove rare actinobacteria is becoming a popular and attractive approach. Even though *Strepotomyces* is the genus producing various types of natural compounds for medical and pharmaceutical purposes, rare actinobacteria isolated from mangrove have a wide range of metabolites with different biological activities. *Micromonospora* is the largest genera among the rare actinobacteria which produces a rich source of natural products such as rifamycin, butemycin and β-carboline. Other genera of the mangrove rare actinobacteria namely, *Jishengella, Salinispora, Saccharopolyspora* and *Nocardiopsis;* exhibited various natural compounds which are potentially useful in the medical and pharmaceutical industry. Previously, one of the most common limitations of conventional metagenomics analysis is restricted taxonomy resolution. The advancement in biotechnology enables the study of strains variation and the dynamic behavior of microbial communities. The NGS technologies enabled the production of results in a rapid, low-cost and reliable manner compared to conventional approaches. These high-throughput sequencing technologies have enabled researchers to discover the microbial diversity from different environments; and also enabled the parallel sequencing of multiple bioactive strains for the identification of secondary metabolites gene clusters through comparative genomics analysis. The knowledge obtained from such studies will be vital for promoting combinatorial biosynthesis of actinobacteria related compounds.

### Conflict of interest statement

The authors declare that the research was conducted in the absence of any commercial or financial relationships that could be construed as a potential conflict of interest.
